# 4,4′-Bipyridine–pyroglutamic acid (1/2)

**DOI:** 10.1107/S1600536809045000

**Published:** 2009-10-31

**Authors:** Hadi D. Arman, Trupta Kaulgud, Edward R. T. Tiekink

**Affiliations:** a Department of Chemistry, The University of Texas at San Antonio, One UTSA Circle, San Antonio, Texas 78249-0698, USA; b Department of Chemistry, University of Malaya, 50603 Kuala Lumpur, Malaysia

## Abstract

In the title co-crystal, C_10_H_8_N_2_·2C_5_H_7_NO_3_, the 4,4′-bipyridine mol­ecule [dihedral angle between the pyridine rings = 36.33 (11)°] accepts O—H⋯N hydrogen bonds from the two pyroglutamic (pga) acid mol­ecules. The pga mol­ecules at each end of the trimeric aggregate self-associate *via* centrosymmetric eight-membered amide {⋯HNCO}_2_ synthons, so that the crystal structure comprises one-dimensional supra­molecular chains propagating in [13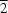]. C—H⋯O and π–π stacking inter­actions [centroid–centroid separation = 3.590 (2) Å] consolidate the structure.

## Related literature

For background to the co-crystallization of active pharmaceutical agents and discussion on the definition of a co-crystal, see: Shan & Zaworotko (2008 ▶); Zukerman-Schpector & Tiekink (2008 ▶). For related studies on co-crystal formation, see: Broker & Tiekink (2007 ▶); Broker *et al.* (2008 ▶); Ellis *et al.* (2009 ▶). For structure analysis, see: Spek (2009 ▶). For hydrogen-bonding considerations, see: Etter (1990 ▶).
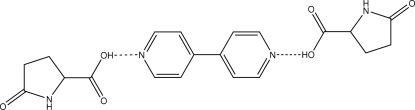


## Experimental

### 

#### Crystal data

C_10_H_8_N_2_·2C_5_H_7_NO_3_
*M* *_r_* = 414.42
Triclinic, 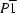

*a* = 7.444 (3) Å
*b* = 11.511 (4) Å
*c* = 12.845 (4) Å
α = 66.274 (17)°
β = 74.203 (17)°
γ = 86.91 (2)°
*V* = 967.6 (6) Å^3^
*Z* = 2
Mo *K*α radiation
μ = 0.11 mm^−1^
*T* = 98 K
0.22 × 0.15 × 0.12 mm

#### Data collection

Rigaku Saturn724 diffractometer
Absorption correction: multi-scan (*ABSCOR*; Higashi, 1995 ▶) *T*_min_ = 0.706, *T*_max_ = 1.000
5618 measured reflections
3375 independent reflections
2851 reflections with *I* > 2σ(*I*)
*R*_int_ = 0.041

#### Refinement

*R*[*F* ^2^ > 2σ(*F* ^2^)] = 0.056
*wR*(*F* ^2^) = 0.128
*S* = 1.16
3375 reflections
281 parameters
2 restraints
H-atom parameters constrained
Δρ_max_ = 0.29 e Å^−3^
Δρ_min_ = −0.26 e Å^−3^

### 

Data collection: *CrystalClear* (Rigaku/MSC, 2005 ▶); cell refinement: *CrystalClear*; data reduction: *CrystalClear*; program(s) used to solve structure: *SHELXS97* (Sheldrick, 2008 ▶); program(s) used to refine structure: *SHELXL97* (Sheldrick, 2008 ▶); molecular graphics: *DIAMOND* (Brandenburg, 2006 ▶); software used to prepare material for publication: *SHELXL97*.

## Supplementary Material

Crystal structure: contains datablocks global, I. DOI: 10.1107/S1600536809045000/hb5194sup1.cif

Structure factors: contains datablocks I. DOI: 10.1107/S1600536809045000/hb5194Isup2.hkl

Additional supplementary materials:  crystallographic information; 3D view; checkCIF report

## Figures and Tables

**Table 1:** Hydrogen-bond geometry (Å, °)

*D*—H⋯*A*	*D*—H	H⋯*A*	*D*⋯*A*	*D*—H⋯*A*
O1—H1*o*⋯N3	0.84	1.75	2.588 (3)	177
O4—H4*o*⋯N4	0.84	1.75	2.582 (3)	175
N1—H1*n*⋯O3^i^	0.88	2.03	2.911 (3)	174
N2—H2*n*⋯O6^ii^	0.88	2.03	2.903 (3)	172
C15—H15⋯O4^iii^	0.95	2.38	3.294 (3)	162
C18—H18⋯O1^iv^	0.95	2.41	3.293 (3)	155

Symmetry codes: (i) 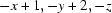; (ii) 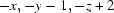; (iii) 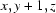; (iv) 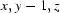.

**Fig. 1.**
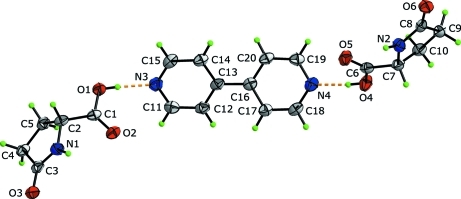
Molecular structure of the asymmetric unit of (I) showing atom-labelling scheme and displacement ellipsoids at the 70% probability level. The O–H···N hydrogen bonds are shown as orange dashed lines.

**Fig. 2.**
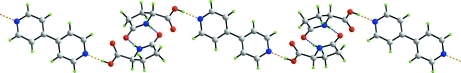
Supramolecular chain formation in (I) mediated by O—H···N (orange dashed lines) and N—H···N (blue dashed lines) hydrogen bonding.

## supplementary crystallographic information

### Comment

The co-crystallization of active pharmaceutical ingredients is an active area of contemporary crystal engineering (Shan & Zaworotko, 2008); see Zukerman-Schpector & Tiekink (2008) for a discussion of terminology. As a continuation of studies into the phenomenon of co-crystallization (Broker & Tiekink, 2007; Broker *et al.*, 2008; Ellis *et al.*, 2009), the co-crystallization of DL-pyroglutamic acid with 4,4'-bipyridine was investigated.

The title co-crystal, (I), comprises two molecules of pyroglutamic acid and one of 4,4'-bipyridine, Fig. 1. The independent molecules of pyroglutamic acid are virtually identical with RMS values for bond distances and angles of 0.006 Å and 0.552 °, respectively (Spek, 2009). The connections between molecules are hydrogen bonds of the type O–H···N, Table 1, in accord with the strongest donor associating with the strongest acceptor (Etter, 1990).

The trimeric aggregates associate into a supramolecular chain *via* eight-membered amide {···HNCO}_2_ synthons. The most convenient description of the chain is given in the following terms. Centrosymmetrically related pyroglutamic acid molecules are connected by the {···HNCO}_2_ synthons and these are bridged by the 4,4'-bipyridine molecules, Table 1 and Fig. 2. The supramolecular chains have a base vector [1 3 - 2] in which alternate 4,4'-bipyridine molecules are connected to pyroglutamic acid molecules of the same chirality.

The chains are consolidated into the 3-D crystal structure by a large number of C–H···O contacts, the shortest two are listed in Table 1, as well as π···π interactions involving both pyridyl rings [the closest *Cg*···*Cg*^i^ = 3.590 (2) Å where *Cg* is the ring centroid of N2, C16—C20 for i: 2 - *x*, -*y*, 1 - *z*].

### Experimental

Colourless crystals of (I) were isolated from the co-crystallization of 1 molar equivalent of DL-pyroglutamic acid (Fluka, 20 mg) and 4,4'-bipyridine (Aldrich, 12 mg) in methanol/ethanol (1/1, 8 ml); m. pt. 425–427 K.

### Refinement

The H-atoms were placed in calculated positions (O–H = 0.84 Å, N–H = 0.88 Å and C–H 0.95–1.00 Å) and were included in the refinement in the riding model approximation with *U*_iso_(H) set to 1.2–1.5*U*_eq_(carrier atom).

### Crystal data

**Table:**

C_10_H_8_N_2_·2C_5_H_7_NO_3_	*Z* = 2
*M**_r_* = 414.42	*F*(000) = 436
Triclinic, *P*1	*D*_x_ = 1.422 Mg m^−^^3^
Hall symbol: -P 1	Mo *K*α radiation, λ = 0.71073 Å
*a* = 7.444 (3) Å	Cell parameters from 3819 reflections
*b* = 11.511 (4) Å	θ = 1.8–40.3°
*c* = 12.845 (4) Å	µ = 0.11 mm^−^^1^
α = 66.274 (17)°	*T* = 98 K
β = 74.203 (17)°	Block, colourless
γ = 86.91 (2)°	0.22 × 0.15 × 0.12 mm
*V* = 967.6 (6) Å^3^	

### Data collection

**Table:**

Rigaku Saturn724 diffractometer	3375 independent reflections
Radiation source: sealed tube	2851 reflections with *I* > 2σ(*I*)
graphite	*R*_int_ = 0.041
Detector resolution: 28.5714 pixels mm^-1^	θ_max_ = 25.0°, θ_min_ = 1.8°
ω scans	*h* = −8→8
Absorption correction: multi-scan (*ABSCOR*; Higashi, 1995)	*k* = −13→13
*T*_min_ = 0.706, *T*_max_ = 1.000	*l* = −11→15
5618 measured reflections	

### Refinement

**Table:**

Refinement on *F*^2^	Primary atom site location: structure-invariant direct methods
Least-squares matrix: full	Secondary atom site location: difference Fourier map
*R*[*F*^2^ > 2σ(*F*^2^)] = 0.056	Hydrogen site location: inferred from neighbouring sites
*wR*(*F*^2^) = 0.128	H-atom parameters constrained
*S* = 1.16	*w* = 1/[σ^2^(*F*_o_^2^) + (0.0416*P*)^2^ + 0.3826*P*] where *P* = (*F*_o_^2^ + 2*F*_c_^2^)/3
3375 reflections	(Δ/σ)_max_ < 0.001
281 parameters	Δρ_max_ = 0.29 e Å^−^^3^
2 restraints	Δρ_min_ = −0.26 e Å^−^^3^

### Special details

**Table:**

Geometry. All s.u.'s (except the s.u. in the dihedral angle between two l.s. planes) are estimated using the full covariance matrix. The cell s.u.'s are taken into account individually in the estimation of s.u.'s in distances, angles and torsion angles; correlations between s.u.'s in cell parameters are only used when they are defined by crystal symmetry. An approximate (isotropic) treatment of cell s.u.'s is used for estimating s.u.'s involving l.s. planes.
Refinement. Refinement of *F*^2^ against ALL reflections. The weighted *R*-factor *wR* and goodness of fit *S* are based on *F*^2^, conventional *R*-factors *R* are based on *F*, with *F* set to zero for negative *F*^2^. The threshold expression of *F*^2^ > 2σ(*F*^2^) is used only for calculating *R*-factors(gt) *etc*. and is not relevant to the choice of reflections for refinement. *R*-factors based on *F*^2^ are statistically about twice as large as those based on *F*, and *R*- factors based on ALL data will be even larger.

### Fractional atomic coordinates and isotropic or equivalent isotropic displacement parameters (Å^2^)

**Table:**

	*x*	*y*	*z*	*U*_iso_*/*U*_eq_	
O1	0.7958 (3)	0.80500 (15)	0.34215 (15)	0.0306 (4)	
H1o	0.8071	0.7262	0.3706	0.046*	
O2	0.7021 (3)	0.76264 (15)	0.20917 (14)	0.0300 (4)	
O3	0.6914 (2)	1.13445 (15)	−0.10317 (14)	0.0250 (4)	
O4	0.6382 (2)	−0.34079 (15)	0.69099 (15)	0.0295 (4)	
H4o	0.6741	−0.2635	0.6605	0.044*	
O5	0.3864 (2)	−0.27410 (15)	0.78866 (14)	0.0278 (4)	
O6	0.0822 (2)	−0.65238 (15)	1.10646 (14)	0.0263 (4)	
N1	0.6307 (3)	1.00631 (18)	0.09308 (17)	0.0239 (5)	
H1N	0.5362	0.9583	0.1002	0.027 (7)*	
N2	0.2248 (3)	−0.51418 (18)	0.91661 (17)	0.0233 (5)	
H2N	0.1380	−0.4596	0.9022	0.028 (7)*	
N3	0.8270 (3)	0.56170 (18)	0.42251 (18)	0.0266 (5)	
N4	0.7503 (3)	−0.10455 (18)	0.60996 (17)	0.0247 (5)	
C1	0.7328 (3)	0.8368 (2)	0.24872 (19)	0.0217 (5)	
C2	0.7041 (3)	0.9777 (2)	0.19365 (19)	0.0215 (5)	
H2	0.6154	1.0033	0.2536	0.026*	
C3	0.7237 (3)	1.1014 (2)	−0.0062 (2)	0.0218 (5)	
C4	0.8716 (3)	1.1608 (2)	0.02102 (19)	0.0228 (5)	
H4A	0.8325	1.2423	0.0263	0.027*	
H4B	0.9921	1.1764	−0.0406	0.027*	
C5	0.8887 (3)	1.0625 (2)	0.1407 (2)	0.0225 (5)	
H5A	0.9011	1.1045	0.1924	0.027*	
H5B	0.9984	1.0116	0.1302	0.027*	
C6	0.4717 (3)	−0.3581 (2)	0.76693 (19)	0.0219 (5)	
C7	0.3977 (3)	−0.4950 (2)	0.82374 (19)	0.0213 (5)	
H7	0.3747	−0.5203	0.7624	0.026*	
C8	0.2141 (3)	−0.6187 (2)	1.0153 (2)	0.0214 (5)	
C9	0.3900 (3)	−0.6883 (2)	0.9955 (2)	0.0236 (5)	
H9A	0.3657	−0.7619	0.9788	0.028*	
H9B	0.4397	−0.7187	1.0655	0.028*	
C10	0.5264 (3)	−0.5884 (2)	0.8884 (2)	0.0248 (5)	
H10A	0.6061	−0.6274	0.8373	0.030*	
H10B	0.6074	−0.5452	0.9133	0.030*	
C11	0.9039 (3)	0.5073 (2)	0.3470 (2)	0.0268 (6)	
H11	0.9653	0.5605	0.2674	0.032*	
C12	0.8973 (3)	0.3770 (2)	0.3805 (2)	0.0243 (5)	
H12	0.9549	0.3417	0.3251	0.029*	
C13	0.8049 (3)	0.2985 (2)	0.4965 (2)	0.0223 (5)	
C14	0.7272 (3)	0.3554 (2)	0.5749 (2)	0.0253 (5)	
H14	0.6659	0.3047	0.6552	0.030*	
C15	0.7406 (3)	0.4857 (2)	0.5344 (2)	0.0273 (6)	
H15	0.6860	0.5234	0.5883	0.033*	
C16	0.7879 (3)	0.1585 (2)	0.5364 (2)	0.0208 (5)	
C17	0.7629 (3)	0.1045 (2)	0.4611 (2)	0.0254 (5)	
H17	0.7590	0.1570	0.3829	0.030*	
C18	0.7440 (3)	−0.0258 (2)	0.5017 (2)	0.0259 (6)	
H18	0.7256	−0.0613	0.4500	0.031*	
C19	0.7758 (3)	−0.0528 (2)	0.6826 (2)	0.0258 (5)	
H19	0.7810	−0.1079	0.7600	0.031*	
C20	0.7947 (3)	0.0765 (2)	0.6494 (2)	0.0242 (5)	
H20	0.8121	0.1093	0.7032	0.029*	

### Atomic displacement parameters (Å^2^)

**Table:**

	*U*^11^	*U*^22^	*U*^33^	*U*^12^	*U*^13^	*U*^23^
O1	0.0477 (12)	0.0234 (9)	0.0292 (9)	0.0069 (8)	−0.0215 (9)	−0.0126 (8)
O2	0.0405 (11)	0.0279 (9)	0.0303 (9)	0.0029 (8)	−0.0146 (9)	−0.0173 (8)
O3	0.0261 (9)	0.0300 (9)	0.0229 (9)	0.0051 (7)	−0.0089 (8)	−0.0135 (7)
O4	0.0262 (10)	0.0242 (8)	0.0340 (10)	−0.0013 (7)	0.0038 (8)	−0.0151 (8)
O5	0.0265 (9)	0.0257 (9)	0.0310 (10)	0.0063 (7)	−0.0029 (8)	−0.0150 (8)
O6	0.0241 (9)	0.0280 (9)	0.0226 (9)	0.0027 (7)	−0.0015 (8)	−0.0093 (7)
N1	0.0239 (11)	0.0249 (11)	0.0243 (11)	0.0001 (9)	−0.0090 (9)	−0.0097 (9)
N2	0.0205 (11)	0.0244 (10)	0.0229 (10)	0.0061 (9)	−0.0055 (9)	−0.0085 (9)
N3	0.0288 (12)	0.0268 (11)	0.0284 (11)	0.0040 (9)	−0.0116 (10)	−0.0133 (9)
N4	0.0194 (11)	0.0269 (10)	0.0277 (11)	0.0034 (9)	−0.0023 (9)	−0.0140 (9)
C1	0.0185 (12)	0.0278 (12)	0.0203 (12)	0.0007 (10)	−0.0030 (10)	−0.0126 (10)
C2	0.0211 (12)	0.0266 (12)	0.0209 (12)	0.0036 (10)	−0.0052 (10)	−0.0144 (10)
C3	0.0222 (13)	0.0230 (12)	0.0252 (13)	0.0081 (10)	−0.0071 (11)	−0.0151 (10)
C4	0.0209 (12)	0.0264 (12)	0.0218 (12)	0.0016 (10)	−0.0013 (10)	−0.0134 (10)
C5	0.0219 (13)	0.0240 (12)	0.0260 (12)	0.0047 (10)	−0.0090 (11)	−0.0132 (10)
C6	0.0229 (13)	0.0264 (12)	0.0183 (12)	0.0031 (10)	−0.0053 (10)	−0.0113 (10)
C7	0.0229 (12)	0.0238 (12)	0.0188 (11)	0.0026 (10)	−0.0045 (10)	−0.0110 (10)
C8	0.0246 (13)	0.0197 (11)	0.0231 (12)	−0.0007 (10)	−0.0072 (11)	−0.0111 (10)
C9	0.0258 (13)	0.0245 (12)	0.0230 (12)	0.0025 (10)	−0.0072 (11)	−0.0120 (10)
C10	0.0252 (13)	0.0263 (12)	0.0255 (12)	0.0031 (10)	−0.0064 (11)	−0.0134 (11)
C11	0.0287 (14)	0.0302 (13)	0.0220 (12)	0.0026 (11)	−0.0090 (11)	−0.0097 (11)
C12	0.0231 (13)	0.0297 (12)	0.0235 (12)	0.0064 (10)	−0.0076 (11)	−0.0140 (11)
C13	0.0198 (12)	0.0268 (12)	0.0250 (12)	0.0032 (10)	−0.0085 (10)	−0.0139 (10)
C14	0.0240 (13)	0.0301 (13)	0.0231 (12)	0.0006 (10)	−0.0040 (11)	−0.0136 (11)
C15	0.0270 (14)	0.0303 (13)	0.0308 (14)	0.0025 (11)	−0.0077 (12)	−0.0189 (12)
C16	0.0164 (12)	0.0250 (12)	0.0219 (12)	0.0019 (9)	−0.0041 (10)	−0.0113 (10)
C17	0.0289 (13)	0.0267 (12)	0.0213 (12)	0.0035 (11)	−0.0056 (11)	−0.0115 (10)
C18	0.0268 (14)	0.0297 (13)	0.0272 (13)	0.0036 (11)	−0.0063 (11)	−0.0183 (11)
C19	0.0246 (13)	0.0293 (13)	0.0220 (12)	0.0001 (11)	−0.0037 (11)	−0.0103 (11)
C20	0.0204 (12)	0.0307 (13)	0.0239 (12)	0.0002 (10)	−0.0038 (11)	−0.0148 (11)

### Geometric parameters (Å, °)

**Table:**

O1—C1	1.314 (3)	C6—C7	1.506 (3)
O1—H1o	0.8400	C7—C10	1.538 (3)
O2—C1	1.213 (3)	C7—H7	1.0000
O3—C3	1.235 (3)	C8—C9	1.514 (3)
O4—C6	1.319 (3)	C9—C10	1.529 (3)
O4—H4o	0.8400	C9—H9A	0.9900
O5—C6	1.213 (3)	C9—H9B	0.9900
O6—C8	1.239 (3)	C10—H10A	0.9900
N1—C3	1.335 (3)	C10—H10B	0.9900
N1—C2	1.449 (3)	C11—C12	1.383 (3)
N1—H1N	0.8800	C11—H11	0.9500
N2—C8	1.338 (3)	C12—C13	1.391 (3)
N2—C7	1.453 (3)	C12—H12	0.9500
N2—H2N	0.8800	C13—C14	1.397 (3)
N3—C15	1.338 (3)	C13—C16	1.482 (3)
N3—C11	1.345 (3)	C14—C15	1.374 (3)
N4—C18	1.332 (3)	C14—H14	0.9500
N4—C19	1.349 (3)	C15—H15	0.9500
C1—C2	1.517 (3)	C16—C20	1.390 (3)
C2—C5	1.550 (3)	C16—C17	1.396 (3)
C2—H2	1.0000	C17—C18	1.377 (3)
C3—C4	1.511 (3)	C17—H17	0.9500
C4—C5	1.533 (3)	C18—H18	0.9500
C4—H4A	0.9900	C19—C20	1.376 (3)
C4—H4B	0.9900	C19—H19	0.9500
C5—H5A	0.9900	C20—H20	0.9500
C5—H5B	0.9900		
			
C1—O1—H1o	109.5	O6—C8—C9	126.3 (2)
C6—O4—H4o	109.5	N2—C8—C9	108.0 (2)
C3—N1—C2	115.02 (18)	C8—C9—C10	104.03 (18)
C3—N1—H1N	125.7	C8—C9—H9A	111.0
C2—N1—H1N	119.2	C10—C9—H9A	111.0
C8—N2—C7	114.5 (2)	C8—C9—H9B	111.0
C8—N2—H2N	127.0	C10—C9—H9B	111.0
C7—N2—H2N	118.5	H9A—C9—H9B	109.0
C15—N3—C11	118.1 (2)	C9—C10—C7	103.70 (19)
C18—N4—C19	117.74 (19)	C9—C10—H10A	111.0
O2—C1—O1	124.3 (2)	C7—C10—H10A	111.0
O2—C1—C2	123.0 (2)	C9—C10—H10B	111.0
O1—C1—C2	112.71 (18)	C7—C10—H10B	111.0
N1—C2—C1	110.12 (18)	H10A—C10—H10B	109.0
N1—C2—C5	103.77 (17)	N3—C11—C12	122.7 (2)
C1—C2—C5	113.37 (19)	N3—C11—H11	118.6
N1—C2—H2	109.8	C12—C11—H11	118.6
C1—C2—H2	109.8	C11—C12—C13	119.0 (2)
C5—C2—H2	109.8	C11—C12—H12	120.5
O3—C3—N1	124.9 (2)	C13—C12—H12	120.5
O3—C3—C4	126.5 (2)	C12—C13—C14	118.0 (2)
N1—C3—C4	108.55 (19)	C12—C13—C16	121.4 (2)
C3—C4—C5	104.45 (18)	C14—C13—C16	120.6 (2)
C3—C4—H4A	110.9	C15—C14—C13	119.3 (2)
C5—C4—H4A	110.9	C15—C14—H14	120.4
C3—C4—H4B	110.9	C13—C14—H14	120.4
C5—C4—H4B	110.9	N3—C15—C14	122.9 (2)
H4A—C4—H4B	108.9	N3—C15—H15	118.5
C4—C5—C2	104.29 (17)	C14—C15—H15	118.5
C4—C5—H5A	110.9	C20—C16—C17	117.6 (2)
C2—C5—H5A	110.9	C20—C16—C13	121.71 (19)
C4—C5—H5B	110.9	C17—C16—C13	120.7 (2)
C2—C5—H5B	110.9	C18—C17—C16	119.2 (2)
H5A—C5—H5B	108.9	C18—C17—H17	120.4
O5—C6—O4	124.4 (2)	C16—C17—H17	120.4
O5—C6—C7	123.4 (2)	N4—C18—C17	123.2 (2)
O4—C6—C7	112.18 (19)	N4—C18—H18	118.4
N2—C7—C6	111.09 (19)	C17—C18—H18	118.4
N2—C7—C10	103.22 (18)	N4—C19—C20	122.7 (2)
C6—C7—C10	114.16 (19)	N4—C19—H19	118.6
N2—C7—H7	109.4	C20—C19—H19	118.6
C6—C7—H7	109.4	C19—C20—C16	119.5 (2)
C10—C7—H7	109.4	C19—C20—H20	120.3
O6—C8—N2	125.7 (2)	C16—C20—H20	120.3
			
C3—N1—C2—C1	−128.8 (2)	C8—C9—C10—C7	−24.8 (2)
C3—N1—C2—C5	−7.2 (3)	N2—C7—C10—C9	23.4 (2)
O2—C1—C2—N1	2.0 (3)	C6—C7—C10—C9	144.11 (19)
O1—C1—C2—N1	−178.66 (19)	C15—N3—C11—C12	−0.1 (3)
O2—C1—C2—C5	−113.8 (2)	N3—C11—C12—C13	1.1 (3)
O1—C1—C2—C5	65.6 (3)	C11—C12—C13—C14	−1.8 (3)
C2—N1—C3—O3	174.8 (2)	C11—C12—C13—C16	178.0 (2)
C2—N1—C3—C4	−5.7 (3)	C12—C13—C14—C15	1.6 (3)
O3—C3—C4—C5	−164.3 (2)	C16—C13—C14—C15	−178.1 (2)
N1—C3—C4—C5	16.1 (2)	C11—N3—C15—C14	0.0 (3)
C3—C4—C5—C2	−19.6 (2)	C13—C14—C15—N3	−0.7 (4)
N1—C2—C5—C4	16.5 (2)	C12—C13—C16—C20	144.2 (2)
C1—C2—C5—C4	135.96 (19)	C14—C13—C16—C20	−36.0 (3)
C8—N2—C7—C6	−136.67 (19)	C12—C13—C16—C17	−36.3 (3)
C8—N2—C7—C10	−13.9 (2)	C14—C13—C16—C17	143.4 (2)
O5—C6—C7—N2	−7.0 (3)	C20—C16—C17—C18	0.8 (3)
O4—C6—C7—N2	173.39 (18)	C13—C16—C17—C18	−178.7 (2)
O5—C6—C7—C10	−123.2 (2)	C19—N4—C18—C17	0.2 (4)
O4—C6—C7—C10	57.2 (3)	C16—C17—C18—N4	−0.7 (4)
C7—N2—C8—O6	178.2 (2)	C18—N4—C19—C20	0.2 (4)
C7—N2—C8—C9	−2.1 (2)	N4—C19—C20—C16	−0.2 (4)
O6—C8—C9—C10	−163.0 (2)	C17—C16—C20—C19	−0.3 (3)
N2—C8—C9—C10	17.4 (2)	C13—C16—C20—C19	179.2 (2)

### Hydrogen-bond geometry (Å, °)

**Table:**

*D*—H···*A*	*D*—H	H···*A*	*D*···*A*	*D*—H···*A*
O1—H1o···N3	0.84	1.75	2.588 (3)	177
O4—H4o···N4	0.84	1.75	2.582 (3)	175
N1—H1n···O3^i^	0.88	2.03	2.911 (3)	174
N2—H2n···O6^ii^	0.88	2.03	2.903 (3)	172
C15—H15···O4^iii^	0.95	2.38	3.294 (3)	162
C18—H18···O1^iv^	0.95	2.41	3.293 (3)	155

Symmetry codes:  (i) −*x*+1, −*y*+2, −*z*; (ii) −*x*, −*y*−1, −*z*+2; (iii) *x*, *y*+1, *z*; (iv) *x*, *y*−1, *z*.
